# Dynamic causal modelling of electrographic seizure activity using Bayesian belief updating

**DOI:** 10.1016/j.neuroimage.2015.07.063

**Published:** 2016-01-15

**Authors:** Gerald K. Cooray, Biswa Sengupta, Pamela K. Douglas, Karl Friston

**Affiliations:** a Wellcome Trust Centre for Neuroimaging, Institute of Neurology, University College London, UK; b Clinical Neurophysiology, Karolinska University Hospital, Stockholm, Sweden

**Keywords:** Dynamic causal modelling (DCM), Bayesian belief updating, Epilepsy, Seizure activity, EEG/ECoG

## Abstract

Seizure activity in EEG recordings can persist for hours with seizure dynamics changing rapidly over time and space. To characterise the spatiotemporal evolution of seizure activity, large data sets often need to be analysed. Dynamic causal modelling (DCM) can be used to estimate the synaptic drivers of cortical dynamics during a seizure; however, the requisite (Bayesian) inversion procedure is computationally expensive. In this note, we describe a straightforward procedure, within the DCM framework, that provides efficient inversion of seizure activity measured with non-invasive and invasive physiological recordings; namely, EEG/ECoG. We describe the theoretical background behind a Bayesian belief updating scheme for DCM. The scheme is tested on simulated and empirical seizure activity (recorded both invasively and non-invasively) and compared with standard Bayesian inversion. We show that the Bayesian belief updating scheme provides similar estimates of time-varying synaptic parameters, compared to standard schemes, indicating no significant qualitative change in accuracy. The difference in variance explained was small (less than 5%). The updating method was substantially more efficient, taking approximately 5–10 min compared to approximately 1–2 h. Moreover, the setup of the model under the updating scheme allows for a clear specification of how neuronal variables fluctuate over separable timescales. This method now allows us to investigate the effect of fast (neuronal) activity on slow fluctuations in (synaptic) parameters, paving a way forward to understand how seizure activity is generated.

## Highlights

• We describe a DCM procedure that provides efficient inversion of seizure activity.
• Similar accuracy but substantially more efficient compared to standard DCM methods.
• Physiological fluctuations over different timescales can be specified.
• This scheme should contribute to understanding seizure activity using DCM.

## Introduction

One of the hallmarks of clinical electroencephalography is the identification of patients with epileptic seizures, where electrographic seizure activity remains one of the most specific and sensitive findings in electroencephalogram (EEG) recordings (Ebersole et al., 2013). Except for a few highly technical applications for source localisation of epileptic activity, clinical EEG recordings are, in essence, still analysed in the same way that they were first analysed by Hans Berger more than a century ago (Ebersole et al., 2013). The main advantage of EEG (and MEG) – compared to other methods of neuroimaging – is the high temporal resolution, allowing for the detection of the rapid neuronal dynamics; including the generation, spread and termination of electrographic seizure activity. However, full use of the information inherent in EEG data requires accurate quantitative methods. This sort of modelling was introduced when the average activities of neuronal populations were first estimated by Wilson and Cowan, using methods from statistical physics, in which individual cells were assumed to follow Hodgkin–Huxley dynamics (Wilson and Cowan, 1972, Wilson and Cowan, 1973). The ensuing neural mass models provide a computationally tractable way of modelling coupled neuronal populations; especially, in the form exemplified by Jansen and Rit (Jansen and Rit, 1995). Neural mass models have been used extensively to characterise the physiology of seizure generation; either in the form of bifurcations (Blenkinsop et al., 2012, Breakspear et al., 2006, Grimbert and Faugeras, 2006, Jirsa et al., 2014, Nevado-Holgado et al., 2012) or multi-stability (Benjamin et al., 2012, Lopes da Silva et al., 2003). In contrast, variation of the model parameters has also been used to model seizures, where inference about these variations calls on Bayesian filtering techniques or genetic algorithms (Blenkinsop et al., 2012, Freestone et al., 2013, Freestone et al., 2014, Nevado-Holgado et al., 2012, Schiff and Sauer, 2008, Ullah and Schiff, 2010, Wendling et al., 2005).

Dynamic causal modelling (DCM) is a very general method which allows for analysis of neural mass models where inferences can be made about the neuronal architectures that underlie measured time series, such as EEG (Stephan et al., 2007). DCM has been widely used in neuroscience in modelling fMRI and EEG activity (David et al., 2006, David et al., 2008, Friston et al., 2012, Moran et al., 2008, Moran et al., 2011a, Moran et al., 2011b, Moran et al., 2011c). DCM rests upon the variational Bayesian inversion of biophysical generative models. Crucially, several models can be inverted for any given data; enabling the evidence for competing models or hypotheses to be evaluated (with Bayesian model comparison). Posterior estimates of the parameters of the winning models then provide a quantitative and physiologically grounded explanation for the observed data.

A phenomenological neural mass model of seizure activity (the Epileptor) has been recently suggested together with a thorough description of bifurcations that give rise to seizures (Jirsa et al., 2014, Proix et al., 2014). Seizure onset and offset are described as saddle node and homoclinic bifurcations, respectively. These sorts of models allow for coupling between dynamics of different time scales, allowing for the transition between ictal and inter-ictal states. From a physiological perspective the coupling between different time scales can be seen as slow fluctuations in synaptic efficacy inducing fast dynamics, and where fast dynamics couple back to synaptic efficacy (Friston, 2014). Pursuing similar ideas – using neural mass models – focal seizure activity was recently modelled using electrocorticography (ECoG) data (Papadopoulou et al., 2015). This dynamic causal modelling study found that changes in intrinsic (within-source) connectivity were required to explain seizure onset and that these slow changes mediated a transient loss of excitatory–inhibitory balance.

Focal epileptic seizures usually evolve through three phases: initiation, propagation and termination (Schiff et al., 2000). During the evolution of the seizure, the spatial dynamics increase in complexity as it is mediated through an increasing cover of an epileptic network, particularly in the propagation and termination phases. Furthermore, seizure activity on EEG recordings is usually in the time scale of minutes or hours. As a result of this, characterising the spatial and temporal evolution of the seizure requires large multiple channel time series. In the context of DCM, this is modelled using large networks of nodes interacting over relatively long periods of time. However, although DCM can be used to model seizure activity over a few minutes, the inversion procedure is computationally expensive and efficient methods for model inversion are required, especially when realistic epileptic networks are inferred. At present batch inversion schemes are used for inversion in DCM and changes in parameters are modelled using mixtures of temporal basis functions. Crucially, all the data are inverted at once, creating a large inverse problem that is relatively expensive to solve. Bayesian belief updating schemes represent an alternative approach that is computationally less intense. Furthermore, it speaks to the physiological context of seizure activity, namely, where the dynamics of fast neuronal activity can be assumed to attain steady state within short epochs of data – while the parameters mediating fast dynamics change slowly from epoch to epoch. This technical note introduces a Bayesian belief updating scheme for the inversion of DCMs of extended time series data that rests upon a separation of fast (neuronal) and slow (synaptic) changes. We test the scheme on simulated as well as real data, and for the latter case compare its accuracy and efficiency with standard Bayesian inversion previously used for DCMs.

## Dynamic causal modelling

In the following DCM analyses, we used a neural mass model to predict electrographic seizure activity. This model represented a modification of the Jansen and Rit model (Jansen and Rit, 1995) and is based on the canonical cortical microcircuit (CMC). It is composed of four subpopulations of neurons corresponding to superficial and deep pyramidal, excitatory, and inhibitory cells (Moran et al., 2013). The subpopulations were interconnected using ten inhibitory and excitatory connections. Afferent connections drove the excitatory granular cells and efferent connections derive from the superficial pyramidal cells. The measurements (ECoG and EEG data) were modelled as a weighted average of the postsynaptic potential of the pyramidal cells. This particular model has been used extensively to model condition-specific changes in synaptic efficacy (in terms of within and between source connectivity) in a number of event related potential studies: e.g., Fogelson et al., 2014, Brown and Friston, 2013, Bastos et al., 2015). Here, we used it to explain the evolution of complex-valued cross spectra produced by seizure activity.

In what follows, we will describe the dynamic causal modelling of successive epochs of data, where neuronal activity is summarised in terms of its spectral density (for a single source) or cross spectral density (for multiple sources). In this paper, we investigate the inferences that can be made on the dynamics of seizure activity when modelling the activity of the seizure onset zone with afferent input from a wider epileptic network. In brief, standard DCM procedures analyse all epochs together, modelling changes in parameters as mixtures of temporal basis functions. This allows one to specify a family of trajectories for slow (epoch to epoch) fluctuations in the model parameters underlying changes in spectral activity that are characteristic of seizure activity. The disadvantage of this approach is that one has to invert a model of multiple epochs, which is computationally very expensive. The alternative is to use Bayesian belief updating, where certain parameters are allowed to change between epochs and others are held constant. Bayesian belief updating essentially updates the priors over parameters for any given epoch based on posterior beliefs, having inverted the previous epoch. We first describe the generic inversion of a single epoch and then consider Bayesian belief updating between epochs.

## Model inversion of a single epoch

In each epoch, the free (synaptic and connectivity) parameters of the neural mass were estimated using DCM for spectral density (Friston et al., 2012, Moran et al., 2011a). This scheme uses variational Bayesian inference under the Laplace approximation (Friston et al., 2007); see Appendix A for a detailed description. Effectively, the model inversion optimises model parameters to maximise Bayesian model evidence, using variational free energy as a proxy for (log) model evidence (Friston et al., 2007). Model evidence (or its lower bound, the free energy) enables the comparison of different models through Bayesian model comparison (Stephan et al., 2010). A difference in log model evidence or free energy of about three constitutes strong evidence for the winning model (with an odds ratio of about 20 to 1). The free parameters together with their prior means and variances are displayed in Table 1.

## Bayesian belief updating

In DCM for cross spectral density (CSD), linear systems theory is used to model the spectral behaviour of seizure activity within each epoch or window. Linear systems theory simplifies the inversion of the model – because the hidden states of the neural mass (state space) model need not be estimated to predict spectral data features – enabling an efficient Bayesian inversion. In brief, the likelihood of the spectral response for a given epoch is computed from the expected spectra for any given model parameters. These neuronal parameters define the system's Jacobian and thereby its implicit transfer functions. These transfer functions are then applied to the (parameterised) spectral density of endogenous neuronal fluctuations. Finally, the expected spectra are generated by adding (parameterised) observation noise. This generative procedure can be expressed formally in terms of a likelihood model for observed sample spectra from the *i*th epoch:

(1) $$g_{i}=\;Η\left(\theta_{i}\right)+\;\xi$$

where Η(*θ*_*i*_) returns the expected spectra for any set of parameters *θ*_*i*_ specifying the transfer functions of the neural mass model – and the spectral density of endogenous fluctuations. This provides the likelihood *p*(*g*_*i*_|*θ*_*i*_) under Gaussian assumptions about sampling error *ξ*.

The variation of time dependent parameters (inhibitory, excitatory connectivity and afferent input) between epochs can now be modelled as a random walk, reminiscent of Markov Chain Monte Carlo (MCMC) samplers (Sengupta et al., 2015a, Sengupta et al., 2015b). In this setting the parameters become the hidden states of a model of (slow) epoch to epoch fluctuations (Särkkä, 2013) and the parametric model can be expressed as an autoregressive (AR) process.

(2) $$\theta_{i}=\;\theta_{i-1}+\varepsilon_{i-1}$$

Here, $\varepsilon_{i}\sim N\left(0,R_{i}\right)$ represents a zero mean Gaussian fluctuation with covariance R_*i*_ (which is parameterised and estimated in each epoch, see Appendix A). This parametric model entails priors about changes in parameters *p*(*θ*_*i*_|*θ*_*i* − 1_). An alternative update scheme would rest on stochastic-approximations (Robbins–Monro or Kiefer–Wolfowitz), where the mean and the covariance matrix are updated based on the Metropolis acceptance criterion (Sengupta et al., 2015a, Sengupta et al., 2015b).

This hierarchical (between epoch) extension of conventional (within epoch) DCM allows us to interpret the implicit Bayesian belief updating as a *prediction step*, where the priors for the current epoch are updated using posteriors over parameters from the previous epoch – and the priors about their changes in Eq. (2):

(3) $$p\left(\theta_{i}|g_{i-1},\ldots ,g_{1}\right)=\int p\left(\theta_{i}|\theta_{i-1}\right)\cdot p\left(\theta_{i-1}|g_{i-1},\ldots ,g_{1}\right)d\theta_{i-1}$$

The subsequent variational inversion using a conventional (within epoch) DCM can be regarded as an *update step*, which uses new data features to provide a new posterior based on the likelihood model in Eq. (1) and the updated priors:

(4) $$p\left(\theta_{i}|g_{i},\ldots ,g_{1}\right)\propto p\left(g_{i}|\theta_{i}\right)\cdot p\left(\theta_{i}|g_{i-1},\ldots ,g_{1}\right)$$

In practice, this means that the priors for the current epoch are taken from the posteriors of the previous epoch.

(5) $$p\left(\theta_{i}|g_{i-1},\ldots ,g_{1}\right)∼\;N\left(\mu_{i-1},Q_{i-1}+\;R_{i}\right)$$

where the previous posteriors are given by $p\left(\theta_{i-1}|g_{i-1},\ldots ,g_{1}\right)∼\;N\left(\mu_{i-1},Q_{i-1}\right)$. This simple form of updating follows because of the Laplace assumption and the form of the autoregressive model above.

The covariance matrices R_*i*_ determine which parameters change and which do not. If a parameter (*θ*_*j*_) does not change over time, then the corresponding variance is zero: {R_*i*_}_*jj*_ = 0. In this instance, the update scheme reduces to standard Bayesian belief updating and the posterior will, usually, converge quickly on the posterior expectation. Heuristically, large prior variances over the parameter fluctuations prevent this convergence and allow for some parameters to perform a random walk over epochs, with varying degrees of smoothness determined by R_*i*_. At each epoch, we estimated the Bayes optimal value of R_*i*_ by parameterising it using R_*i*_ = (1 + η_*i*_)R_0_ and optimising η_*i*_ with respect to free energy (this new parameter is a volatility parameter): see Appendix A for details. A simpler scheme would assume a fixed volatility R_*i*_ = R_0_ however, optimising the volatility allows one to quantify periods of greater fluctuations, at little computational expense – as we will illustrate below.

The corresponding smoothness of fluctuations in time-varying or volatile parameters in standard schemes is enforced by using temporal basis functions. This affords a more constrained model of parametric fluctuations that may or may not be appropriate, for example, when there are large transitions around seizure onset. In this sense, the standard approach is more constrained with fewer free parameters; namely, the coefficients of the temporal basis set (in what follows, a set of 8 discrete cosine functions for each volatile parameter). In contrast, the number of free parameters for the Bayesian update scheme corresponds to the number of epochs for each parameter; however, priors on their transitions suppress their effective degrees of freedom. Any temporal basis set could be used, however, for simplicity and their well-behaved boundary conditions, we used a set of cosine functions. For the data modelled in this study using a larger set (> 8) of discrete cosine functions did not change the estimated parameters substantially.

In summary, the Bayesian belief updating scheme estimates slowly varying parameters as follows:

• Window the data into suitable epochs.
• Estimate the posteriors of the parameters in the first epoch.
• Replace the priors over the parameters in the next epoch with the posteriors of the previous.
• Estimate the posteriors of the parameters in the next epoch.
• Continue recursively for all epochs.

The standard update system estimates volatile parameters as follows:

• Window the data into suitable epochs.
• The trajectory of each parameter is parameterised using a set of smooth temporal basis functions (e.g. discrete cosine set).
• Estimate the posteriors over the coefficients of the basis set that describe the smooth variation of the original parameters (i.e., estimate the parameterised trajectory of the neuronal parameters).

## Model inversion

### Simulated EEG data set

**Fig. 1:**
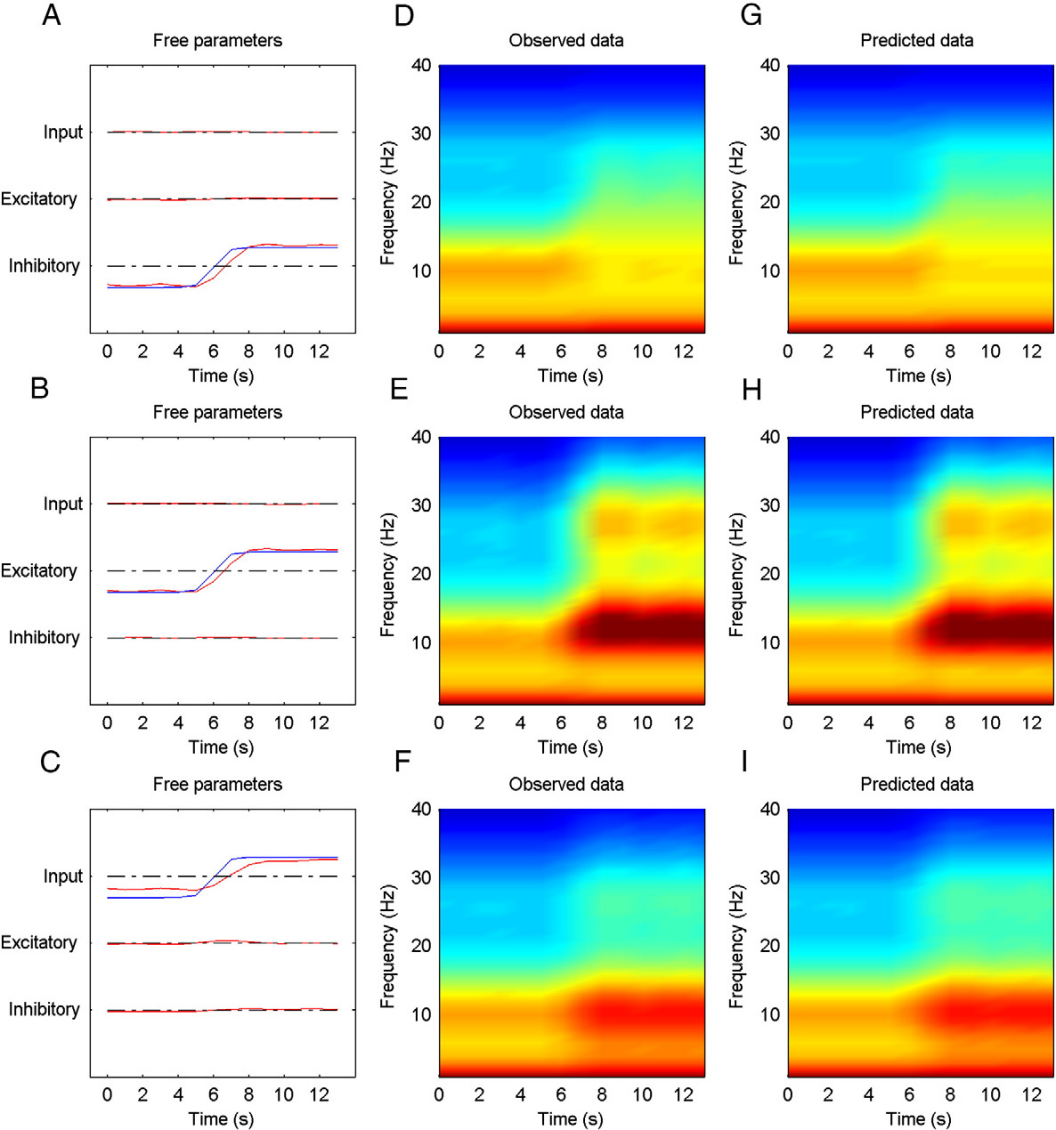
The panels to the left (A, B, C) show the known and predicted changes in inhibitory, excitatory and endogenous input to a simulated cortical source (blue simulated changes and red inferred changes). The fields in the middle (D, E, F) and right (G, H, I) column represent the observed and predicted time frequency response of the simulated data.

In this section, we will compare the inferences about changes of intrinsic connectivity and endogenous input for simulated data with the actual drifts of these parameters. We simulated data that represented local field potentials close to a region of the cortex undergoing slow changes (< 0.5 Hz) in the intrinsic (excitatory and inhibitory) connectivity or endogenous input. The data consisted of 14 s of activity recorded with one channel. Three simulations were performed with drifts of inhibitory, excitatory or endogenous input. The parameters were changed using a sigmoid function of time, increasing from 0 to 1 during the 14-s time period. The simulated data was inverted using the Bayesian belief updating scheme described above. Reassuringly, we were able to recover parametric fluctuations very similar to the true changes for all (three) types of parameters. Please see Fig. 1.

### Empirical EEG/ECoG data set

To establish validity of the Bayesian belief updating, we compared the belief updating and temporal basis functions schemes, when applied to real data. In brief, we used model comparison to optimise prior beliefs parametric fluctuations and compared the Bayesian updating scheme with the standard (basis functions) approach. We examined inference about models and parameters, qualitatively and quantitatively. Given that the priors on parametric fluctuations are formally distinct between the two schemes (a mixture of temporal basis functions versus a random walk), we hoped to establish construct validity between the two schemes in terms of the preferred models and their physiological implications.

Model comparison was performed for each data set, comparing eight models which allowed for different combinations of the three different types of neuronal parameters (inhibitory, excitatory connectivity and the parameters of the power law spectral density of endogenous afferent input) to change over time. This was compared for both types of inversion schemes – (Bayesian belief) updating and standard (basis function) inversion.

**Fig. 2:**
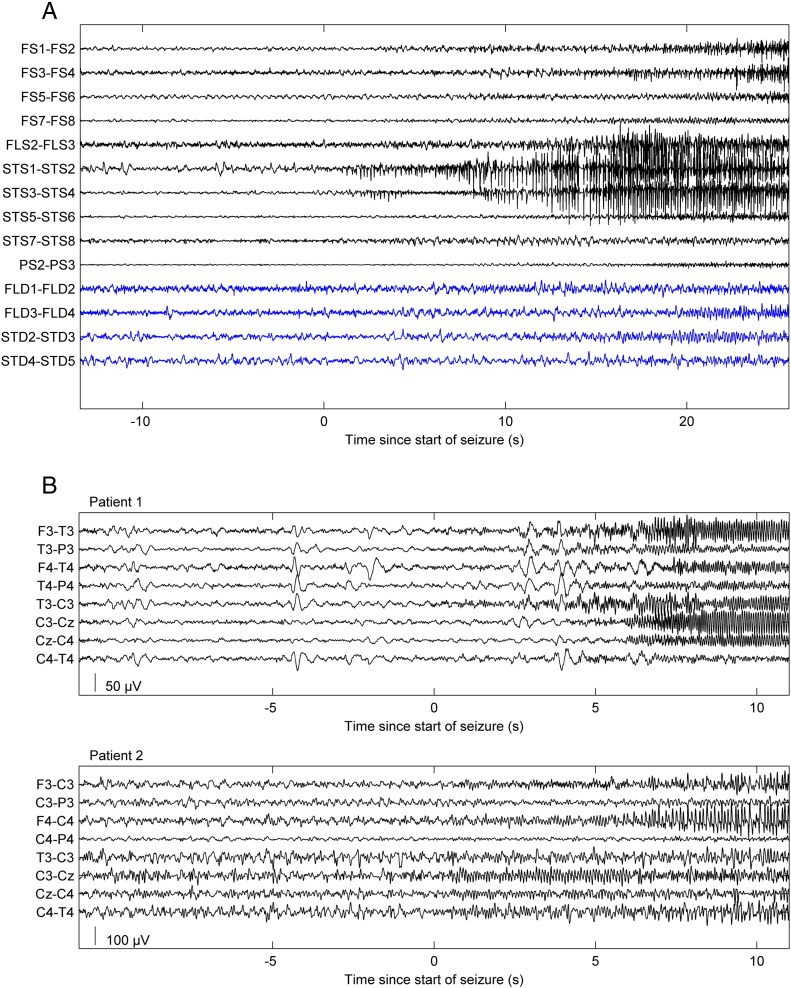
A) Seizure activity recorded using ECoG. Note the focal start of seizure with relatively rapid spreading between electrodes. Electrodes in black are located on the right temporal lobe and blue electrodes over the left temporal lobe. B) Seizure activity recorded using EEG from two patients. Note the focal start of seizure with relatively quick spreading between electrodes.

Electrocorticogram (ECoG) data were collected retrospectively from the database at Clinical Neurophysiology at Karolinska University Hospital, Stockholm, Sweden and comprised invasive ECoG from a patient with focal temporal lobe epilepsy. Data was registered using 32 electrodes placed over the temporal lobes bilaterally. The seizure onset zone was located to the lateral temporal lobe on the right side, where we identified the onset of electrographic seizure activity, see Fig. 2A. We modelled the activity of the seizure onset zone during seizure onset, spread and termination. We used eleven seizures that were free of artefacts for modelling the averaged induced spectral activity. After acquisition, the data was filtered using a bandpass filter (Butterworth 5th order filter) between 0.5 and 70 Hz. Line activity was removed using a notch filter at 50 Hz. The time series for each seizure was divided into 2000 ms epochs without overlap. The size of the window was chosen as the maximum duration over which spectral activity remained approximately constant. More specifically, we used the maximum window length that retained 90% of the spectral power (as estimated using a complex Gaussian wavelet).

EEG recordings were obtained retrospectively from two patients (EEG data set 1 and 2) with recurrent partial seizures from the database at Clinical Neurophysiology at Karolinska University Hospital, Stockholm, Sweden. These patients were clinically determined to have anti-N-methyl-D-aspartate receptor (NMDA-R) antibody encephalitis. The EEG recording comprised nine scalp electrodes positioned according to the 10–20 system (F3, F4, C3, C4, Cz, P3, P4, T3 and T4) together with a reference electrode placed over Fz. Seizure activity from both patients started with fast activity in the beta and alpha band, which increased in amplitude and was accompanied by a reduction of oscillatory frequency, before terminating, see Fig. 2B. The duration of the seizures was approximately 15 and 60 s respectively in EEG data set 1 and 2. A total of 55 seizures free of artefacts were selected for modelling from EEG data set 1 and two seizures were selected from EEG data set 2. After acquisition, the data was re-referenced to a common average and filtered using a bandpass filter (Butterworth 5th order filter) between 0.5 and 70 Hz. Line activity was removed using a notch filter at 50 Hz. We used an empirical Bayes beamformer to locate the source with the greatest spectral power during the first second of seizure activity in each patient (Belardinelli et al., 2012). We then used the reconstructed source activity at this location for further analysis. The time series for each seizure was divided into 2000 ms windows without overlap, for both patients. The size of the epoch was chosen using the same criteria applied to the invasive data described above. Based upon the resulting spectral density of seizure activity for both ECoG and EEG data, we modelled fluctuations in spectral power between 1 Hz and 40 Hz with DCM.

### Model comparison

We compared eight models that allowed for different combinations of the three different sets of parameters to fluctuate: inhibitory, excitatory and the parameters of (the power law) spectral density of afferent input. Furthermore, we parameterised the noise process and estimated it by maximising the free energy (using an autoregressive process of order 1). The model with volatile inhibitory and excitatory connectivity and spectral input showed highest evidence for all of the three data sets. The best model explained more than 98% of the variation of the data for both types of seizure recordings, invasive and non-invasive. We obtained the same results with the standard inversion of seizure data, giving similar inferences on the causal dependence of modelled seizure activity to afferent input from un-modelled parts of a wider epileptic network. The free energy for each model inversion is shown in Table 2 with respect to the free energy of the winning model.

### Variation in synaptic efficacy

**Fig. 3:**
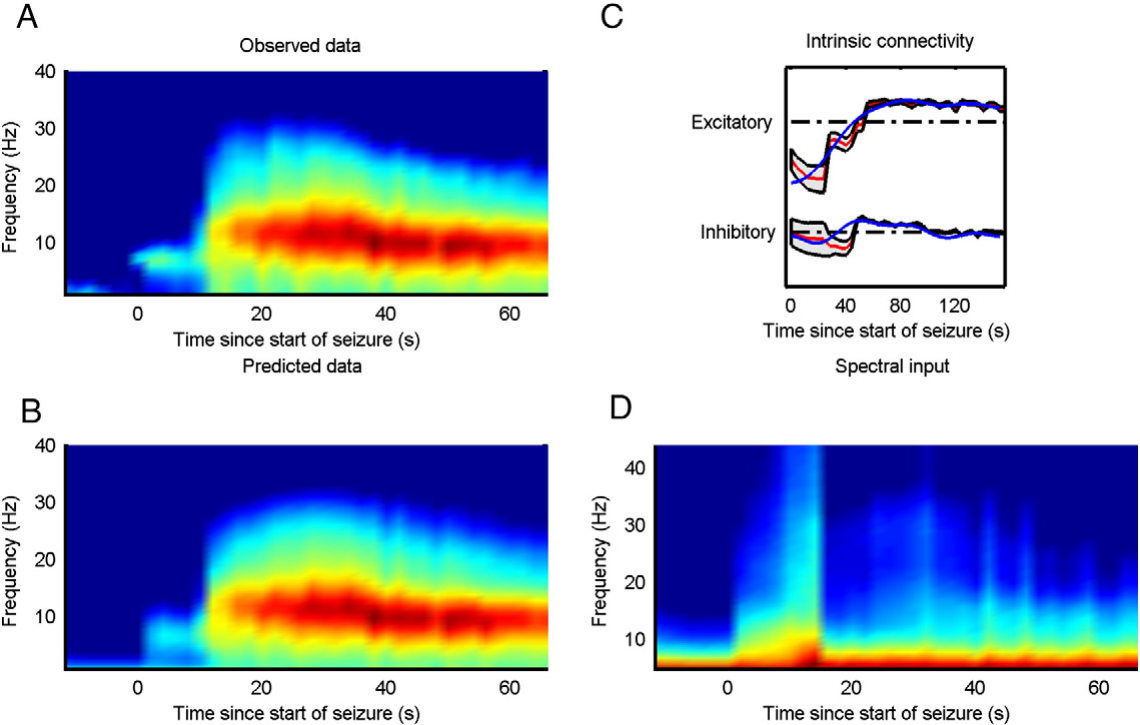
The panels to the left (A, B) show the observed and predicted time frequency activity of the seizures recorded using ECoG. Top right (C) shows estimated changes in red using the updating scheme and the corresponding changes estimated using the standard scheme in blue. The (~ 95%) confidence interval of estimated parameters (± 2 standard deviations) is indicated by the grey area encompassing the posterior expectation (red curves). Bottom right (D) shows the inferred spectral input to the modelled cortical source using the updating scheme.

**Fig. 4:**
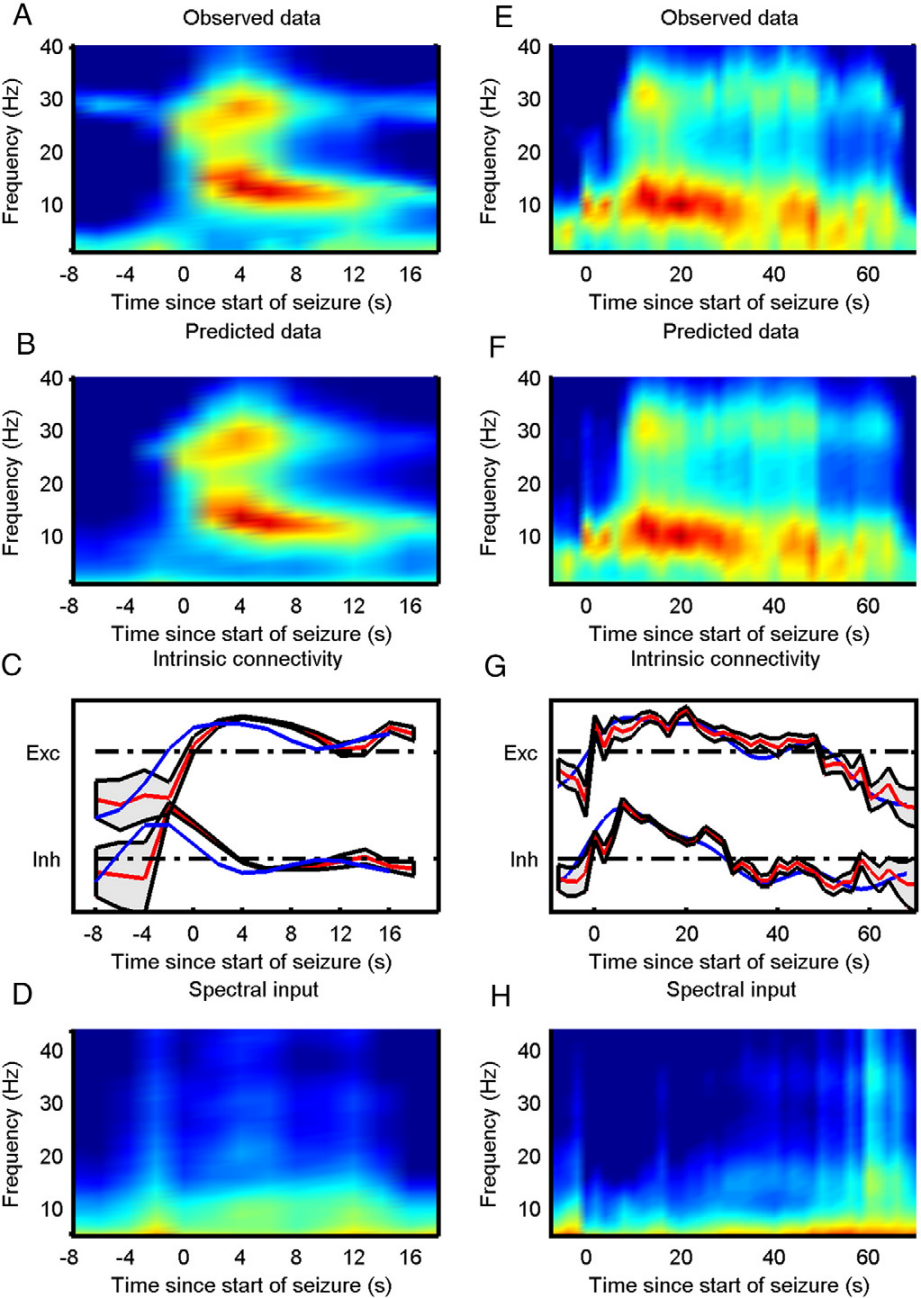
Panels to the left (A, B, C, D) show results from EEG data set 1. Panels to the right show results from EEG data set 2 (E, F, G, H). Top row (A, E) shows observed data. Second row (B, F) shows the predicted activity after model inversion. Third row (C, G) shows the inferred changes in intrinsic connectivity where red is used for inferences from the updating scheme and blue reports the corresponding changes estimated using the standard scheme. The (~ 95%) confidence interval of estimated parameters (± 2 standard deviations) is indicated by the grey area encompassing the posterior expectation (red curves). The bottom row (D, H) shows the inferred spectral input to the modelled cortical region using the updating scheme.

**Fig. 5:**
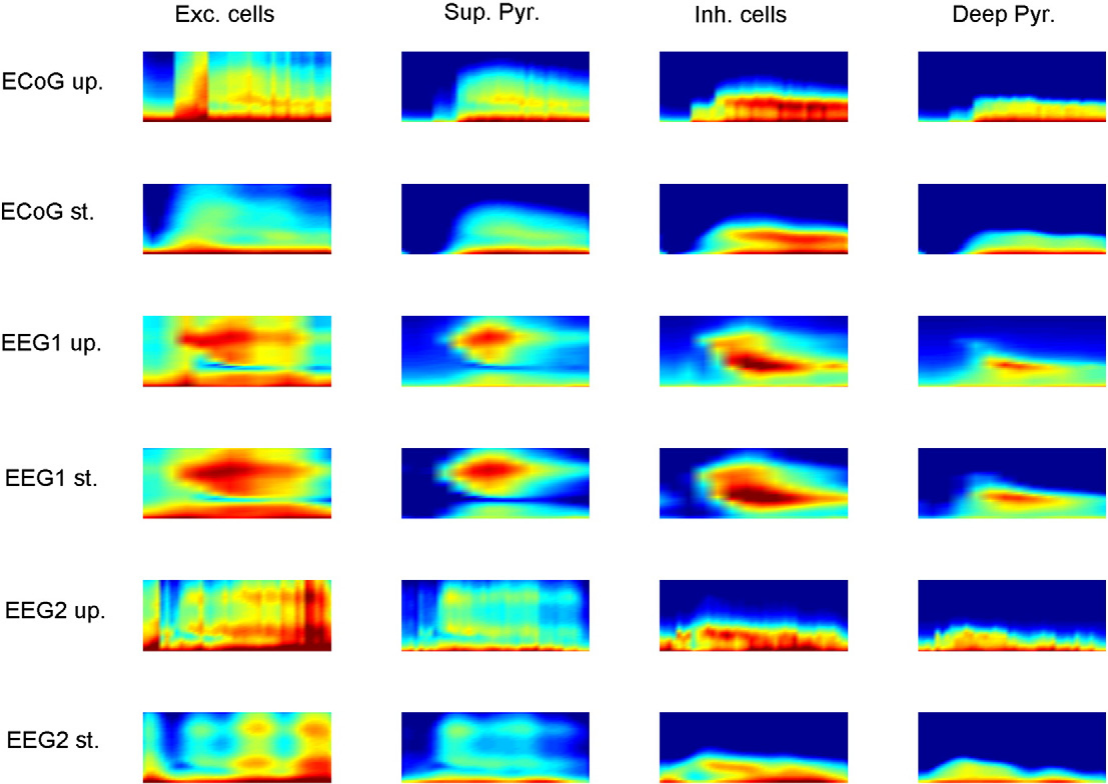
The top row shows the inferred time frequency activity of the four subpopulations (excitatory cells, superficial pyramidal cells, inhibitory cells and deep pyramidal cells) using ECoG data and the updating scheme (ECoG up.). The second row shows the same results but using the standard scheme (ECoG st.). The third row shows inferred time frequency activity of the four subpopulations using EEG data set 1 and the updating scheme (EEG1 up.). Fourth row shows the same but using the standard scheme of inversion (EEG1 st.). The fifth row shows the inferred time frequency activity of the four subpopulations using EEG data set 2 and the updating scheme (EEG2 up.). Sixth row shows the same but using the standard scheme of inversion (EEG2 st.).

**Fig. 6:**
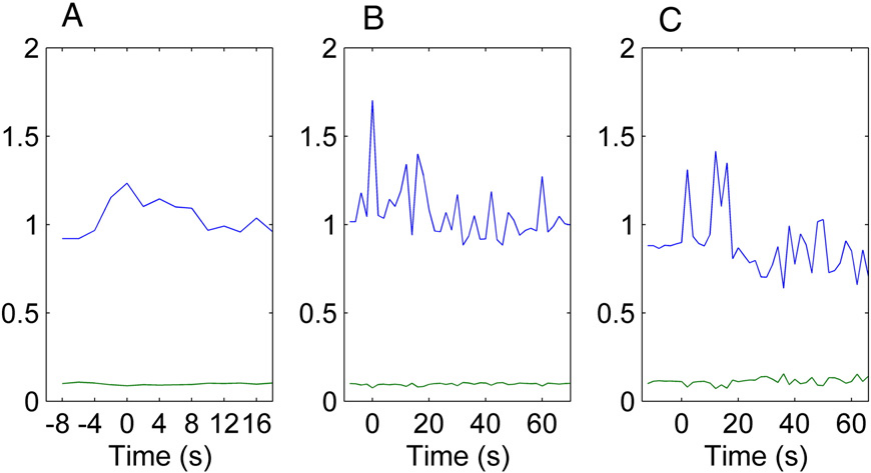
(A) Estimated noise process for EEG data set 1 and (B) results for EEG data set 2. (C) Estimated noise process for the ECoG data. The mean of the volatility parameter is depicted in blue and its variance in green.

The winning model showed similar changes in both inhibitory and excitatory connectivity between the two inversion schemes for all three sets of seizure data. See Fig. 3, Fig. 4 for the observed data and posterior estimates for the two types of data. To further characterize the effect of these changes on the four populations of neurons in the epileptogenic source, we estimated the spectral activity of each population, under the expected parameters of the best model. This reconstruction of hidden neuronal activity revealed similar changes for both inversion schemes, although the smoothness of the requisite inversion was not always as evident for the updating scheme, see Fig. 5. In Table 3, we compare the fits of the model to the data using the two inversion schemes. It was clear that both inversions manage to explain more than 95% of the variance in all three sets of data. Furthermore, in all three cases the volatility of the parametric fluctuations increased prior to seizure onset, at the point of changes in spectral activity, see Fig. 6. See Appendix A for a detailed description of how this volatility parameter was updated.

### Computational efficiency

The inversion schemes were computed on a Dell Precision T3610 (3.5 × 4 GHz; 16 GB random access memory). The updating schemes were computationally less expensive, being 10 to 20 times faster. Inversion of the winning model for both invasive and non-invasive data took approximately 5 min compared to approximately 1–2 h when using the standard inversion scheme.

## Discussion

This technical note presents a computationally efficient scheme within which the cortical physiology of epileptic seizure activity can be inferred using invasive or non-invasive electrophysiological recordings. The method rests upon the assumption that the physiology of epileptic seizures takes place on at least two time scales. This has been verified analytically, numerically, *in vitro* and *in vivo* (including humans) in several studies (Bernard et al., 2014, Friston, 2014, Jirsa et al., 2014 Papadopoulou et al., 2015, Proix et al., 2014, Wendling et al., 2002, Terry et al., 2012), where the transition between inter-ictal and ictal states can be viewed as a coupling between slow and fast dynamics. This coupling can be modelled using parameters that generate fast activity but vary themselves on a slower time scale, the fast part describing the spectral output of the electrographic seizure and the second its waxing and waning over time. From an inference point of view, this leads to a (adiabatic) simplification in the estimation of the parameters, where efficient updating techniques can be used to estimate slow variables. In the present study, we used a Gaussian random walk for the slowly varying or volatile parameters. By estimating the covariance (i.e., volatility) of the parameter fluctuations, we were able to estimate their fluctuations over time using Bayesian model comparison. This is similar to sampling based adaptive-MCMC schemes, where the parameter variation is learnt online based on an acceptance criterion (Sengupta et al., 2015a).

The inference scheme described above assumes that the cortex reaches a quasi-steady state before the synaptic parameters or the external input changes significantly. A long window of observation of the steady state will allow for an accurate estimation of the underlying parameters governing the dynamics. However, often the window length of observation will need to be relatively short due to the drift of the parameters (quasi-steady state and not true steady state) but averaging the spectral activity over several instances of seizure activity should give reliable estimates of the underlying parameters. This averaging of spectral activity over several identical seizures furnishes a robust method for estimating the parameter trajectories underlying a specific seizure type. The identification of similar (clinical) seizures can be based on the clinical analysis of the EEG or ECoG data and the clinical symptoms. However, the inference scheme also provides confidence bounds on the estimated parameters, which provides information on the uncertainty of the inversion. In Fig. 3, Fig. 4 confidence bounds are shown around the mean parameter trajectory. We have also included the parameter variations estimated using the standard DCM inversion scheme for comparison.

Similar methods using random walks to infer parameter fluctuations have been described previously, using different filtering schemes (Freestone et al., 2013, Freestone et al., 2014, Schiff and Sauer, 2008, Ullah and Schiff, 2010). These methods invert signals in the time domain, in contrast to the frequency (spectral) domain used in the present approach. Inverting spectral activity generally leads to a simplification (e.g., linearization) of the model, which might be valid in certain cases. Regarding seizures, there are instances when the phase of the signal is of less importance than the amplitude of oscillatory activity. Indeed amplitude differences or variation in oscillatory frequency generally constitute the clinical criteria that define electrographic seizure activity (Young et al., 1996). However, in other cases, the phase of the signal may play an important role in characterising seizure activity; e.g., 3 Hz spike and wave, poly-spike and wave, spike and slow wave activity. For these cases, the method presented here may miss important features of the electrographic seizure activity, while inference in the time domain would retain these features. Moreover, parameter estimation in the spectral domain, using the method we describe, assumes linearity (i.e. the non-linear ordinary differential equation representing the neural mass model is approximated by its linearization). The main motivation for this approximation is the ensuing simplification of the inversion scheme, resulting in relatively quick inversions, compared to more accurate inversion schemes that include the non-linearities. The non-linearity of the neural mass models we used lies in the sigmoidal function that relates membrane potential to average firing rate: see Freestone for a description of how this sigmoidal function affects Bayesian inversions schemes (Freestone et al., 2014). It is interesting to compare the (spectral) scheme presented here with time domain methods: this distinction determines which types of seizures could be inverted under the assumption of stationarity and mild non-linearity (present model) by comparing with results obtained from models where these assumptions are relaxed (see Freestone et al., 2013, Freestone et al., 2014, Schiff and Sauer, 2008, Ullah and Schiff, 2010). In future studies, we will compare (spectral and filtering) DCM schemes with related methods (Freestone et al., 2014, Schiff and Sauer, 2008, Ullah and Schiff, 2010).

In the current model, we did not consider any coupling of the fast activity to the slowly varying parameters, the variation was completely data driven; however, inclusion of this coupling (i.e. activity dependent plasticity) may improve our estimates of the slow parameters – and allow us to predict their future values.

Interestingly, such coupling between slow and fast fluctuations is a feature of gradient-based MCMC sampling schemes, where the scale dependence is used to boost computational efficiency (Sengupta et al., 2015b). The combination of the updating method presented in this paper, together with a suitable forward model could provide an efficient variational scheme for more precise inference on seizures dynamics. A phenomenological model was presented in the Epileptor model, where a slow parameter was introduced with a reciprocal coupling to fast dynamics (Jirsa et al., 2014). We will, in future, introduce a biologically plausible forward model for the slow parameters, similar in form to the forward model in the Epileptor, which might allow for improved estimation of slow parameters.

One advantage of using a biologically plausible model for prediction of seizure dynamics is the possibility of implementing directed therapeutic interventions. In the dynamic causal models used for this study, the equations governing the dynamics describe, to some approximation, neurobiological quantities. The hidden states of the model represent population averages of postsynaptic potentials and currents, while the parameters represent the synaptic connectivity between neuronal populations. The slow variation of the parameters can be seen as variation of synaptic gain. This could be due to various biophysical and biochemical causes such as membrane potential dependent conductivity of ion channels or changes in ion concentrations. As epileptic seizures are sometimes prolonged – and can last for minutes or hours – this opens the possibility for metabolic changes in receptors. Furthermore, seizures are not physiological events and as such may affect the dynamics by more deleterious effects such as energy or oxygen depletion (Sengupta and Stemmler, 2014). All of the changes in synaptic connectivity described above could affect the way the slow parameters change and are candidates for a forward model.

The accuracy of the Bayesian belief updating scheme proved in general to be robust – giving similar results to standard DCM schemes – but also correctly inferring variations in parameters in simulated data. Inspection of the results from both inversion schemes led to similar conclusions but the quantitative descriptions were slightly different. This indicates that some predictions may only confer qualitative and not quantitative information. The updating scheme only considers data sampled prior to the estimation of the current parameter (technically, a filtering scheme), which is a less constrained inverse problem than conditioning the parameters on all the data (a smoothing scheme), which is also a feature of the standard DCM inversion scheme. However, the similarity between the inversions using the two schemes presented in this note indicates that convergence and local minima problems might not be much greater for the updating scheme, relative to the standard scheme. Moreover, combining these schemes (belief updating and the standard scheme) may also address issues pertaining to local minima – where multiple re-starts are generally assumed to be the only solution.

We studied both invasive and non-invasive data, inferring changes in both synaptic efficacy and afferent input to the cortical source. It is interesting that we were able to infer these changes using non-invasive data. This required localising the seizure activity first – for which we used a beamformer approach. However, the non-invasive recordings are confounded by several unknown physical properties of the head and also measured at several orders of magnitude away from the seizure activity zone compared to the invasive recordings. The source localisation used to accommodate these unknowns may itself introduce errors into the modelling, especially if the region generating seizure activity consists of extended or multiple sources. If spatial dynamics were included in our model we would have most probably seen a greater difference between the type of inferences that we make using the two types of data. In the present model, the spatial dynamics are collapsed into the afferent input to the modelled source. The inversion scheme did allow us to estimate which part of the seizure activity was due to afferent input; however, we cannot say anything regarding the physiology of the endogenous inputs using the current model.

At present, we have not been able to validate our scheme using e.g. more invasive methods, where the intrinsic dynamics can be measured; however, the DCM used for this study has been validated in previous studies using local field potential recordings, together with pharmacological manipulations or with micro-dialysis measurements of extracellular glutamate levels (Moran et al., 2011a, Moran et al., 2011c). Moreover, invasive measurement of subpopulation activity could be used for partial validation, as we can infer these values from the data. Further studies, with the possibility of validating the method and model presented in this study would be useful for future application of DCM in epilepsy research and might have clinical implications.

The parametric model used in belief updating allows synaptic efficacy (and afferent input) to perform a random walk during seizure activity. The rate of change of these parameters over time is governed by the underlying noise process. Physiologically this would describe the effects of un-modelled processes on the parameters. A volatile noise process would represent large influences on the synaptic efficacy and afferent input, whereas a small amplitude noise process would represent a small random influence on the parameters. To summarise, the noise process does not model a specific physiological process but instead estimates the size of un-modelled physiological processes, which can be estimated by maximising the free energy of the problem (see Appendix A). In the present model we did see an increase in volatility when the spectral features of the seizure changed, which might indicate that – at seizure onset – there is an unknown effect on parametric fluctuations, which is itself volatile. Future investigation using more elaborate (e.g., hierarchical) models could be of value, as they might explain changes in volatility and hence furnish a better understanding of the dynamical pathophysiology of seizure activity.

We have considered the waxing and waning of seizure activity, and modelled two time scales over which the dynamics evolved. However, epileptic seizures cluster over time (Osorio et al., 2009, Tauboll et al., 1991). The inter-seizure interval has been described as a power law, as has the probability of seizure intensity (Osorio et al., 2009). These behaviours indicate that seizures may have scale free features. Events with scale free behaviour are known to occur close to transition points of dynamical systems – and are associated with the notion of self-organised criticality (Bak et al., 1987). It might be that the cortical dynamics generating epileptic seizures have many characteristics of a dynamical system close to a bifurcation, as is implemented explicitly in the Epileptor model (Jirsa et al., 2014). The interaction between two time scales involved in seizure dynamics might be a feature of a more widespread interaction between multitudes of timescales. The model described in this study can, with some modifications, accommodate several interacting timescales. This can be done by including a hierarchy of parameters each with its own timescale, as with the volatility parameter. The timing between seizures (inter-seizure interval) might be governed by parameters changing at an even slower time scale than the synaptic gains. Several Bayesian methods could be used for estimating dynamic parameters in hierarchical systems such as hierarchical Gaussian filtering and switching mesostate space models (SMSM) (Mathys et al., 2011, Olier et al., 2013). Furthermore, SMSM would also resolve the spatial dynamics of electrographic seizure activity recorded non-invasively.

The updating scheme proved very efficient compared to the standard DCM inversion as computation times improved approximately 10–20 fold. This increase in efficiency might be due to the decoupling of epochs, together with a computationally cheap estimation of the transition of means and covariances. This form of inversion may be useful when modelling realistic epileptic networks with large numbers of nodes. Augmented with efficient gradient/curvature estimators (Sengupta et al., 2014), model comparison (an important aspect of DCM analysis) could then be entertained over relatively large model spaces, as each inversion could be completed fairly quickly.

In conclusion, we have implemented a Bayesian belief updating scheme, by splitting the dynamics of seizures into fast and slow parts, to make inferences about the slow changes in synaptic efficacy and afferent input causing epileptic seizure activity. There are two benefits of this implementation compared to standard DCM inversion schemes. First, the set of equations modelling neuronal dynamics is separated into two physiological components with fast (neuronal) and slow (synaptic) hidden variables. In this setting, it is easier to understand the coupling of variables fluctuating over separable timescales, which is a key feature of seizure activity (Friston, 2014). Secondly, there is a clear reduction in computational cost in performing the inference, which may be important when applying these methods to large data sets from patients with seizures.

## Funding

This work was supported by the Wellcome Trust (B.S. and K.J.F; 088130/Z/09/Z) and a postdoctoral scholarship from the Swedish Brain Foundation (Hjarnfonden) to G.K.C. P.D. was supported by the Klingenstein Foundation, and the Keck Foundation.

## Figures and Tables

**Table 1:** (Free) Parameters estimated by dynamic causal modelling. The second column describes the prior values and the third the log variances.

Parameters estimated			
Constant parameters	Notation	Prior mean	Log prior variance
Time constants (Hz)	*T_i_* , *i* = 1, …, 4	[0.25 0.17 0.08 0.07] * 1000	0.0625
Connectivity constants (Hz)	*g_i_*, *i* = 1, 2, 7, 10	[0.8, …, 0.2] * 1000	0.0625
Slope of sigmoid function	*γ*	0.67	0.03125
Time delay for connections (ms)	*d*	1	0.03125
*Time dependent parameters*			
*Connectivity parameters*			
Inhibitory (Hz)	*g*_3_(*t*)	1.6 * 1000	0.0625
Inhibitory (Hz)	*g*_4_(*t*)	0.8 * 1000	0.0625
Inhibitory (Hz)	*g*_9_(*t*)	0.4 * 1000	0.0625
Excitatory (Hz)	*g*_5_(*t*)	0.8 * 1000	0.0625
Excitatory (Hz)	*g*_6_(*t*)	0.4 * 1000	0.0625
Excitatory (Hz)	*g*_8_(*t*)	0.8 * 1000	0.0625

*Endogenous spectral input*			
Amplitude of spectral density of input	*a*_1_(*t*)	1	0.0078125
Power law exponent of spectral density of input	*a*_2_(*t*)	1	0.0078125
Amplitude of spectral density of measurement noise	*b*_1_(*t*)	1	0.0078125
Power law exponent of spectral density of measurement noise	*b*_1_(*t*)	1	0.0078125
Spectral innovation of input	*d_i_*(*t*), *i* = 1, …, 8	1	0.0078125

**Table 2:** The variance explained and the free energy for the different models inverted for the different types of inverted models. Note that the winning model (highest free energy, in bold) also had the best fit. The free energies are expressed relative to the null model.

Model	ECoG data 1		EEG data 1		EEG data 2	
	Variance explained	Free energy	Variance explained	Free energy	Variance explained	Free energy
Inhibitory + excitatory + endogenous	**0.99745**	**2.33E + 04**	**0.98425**	**4.11E + 03**	**0.9949**	**1.50E + 04**
Inhibitory + excitatory	0.99212	2.20E + 04	0.94117	3.31E + 03	0.9705	1.30E + 04
Inhibitory + endogenous	0.99621	2.26E + 04	0.94503	3.41E + 03	0.99312	1.45E + 04
Excitatory + endogenous	0.98931	2.16E + 04	0.97685	3.69E + 03	0.98529	1.40E + 04
Inhibitory	0.28691	3.02E + 02	0.22256	-4.29E + 02	0.65903	1.49E + 03
Excitatory	0.95	1.69E + 04	0.86233	2.47E + 03	0.90751	9.93E + 03
Endogenous	0.98875	2.13E + 04	0.92813	3.04E + 03	0.98418	1.38E + 04
Null	0.28658	0.00E + 00	0.36074	0.00E + 00	0.5915	0.00E + 00

**Table 3:** The variance explained of the data for the optimal full model with two schemes of data inversion, differences less than 0.05.

Model	ECoG data 1		EEG data 1		EEG data 2	
	Updating	Time basis	Updating	Time basis	Updating	Time basis
Inhibitory + excitatory + endogenous	0.997	0.987	0.984	0.981	0.995	0.957

## Appendix A

### A.1 Variational Laplace

#### A.1.1 Summary

The detailed derivations below provide the mathematical background to the algorithms used in this study. They describe how variational free energy is used as a proxy for model evidence – and it is estimated, for any generative model. When the parameters of the model are changed, the free energy will change accordingly. See details below.

#### A.1.2 Variational Laplace

For more details, see Friston et al. (2007) and Mathys (201)). A (dynamic causal) generative model can be characterised with the joint probability distribution of the parameters/states of the model and data, *p*(*y*, *θ*). The evidence for the data given the model can be expressed as follows:

(A.1) $$\ln p\left(y\right)=\int d\theta \left[q\left(\theta \right)\ln p\left(y,\theta \right)-q\left(\theta \right)\ln q\left(\theta \right)\right]+D_{KL}\left(q\left(\theta \right)||p\left(\theta |y\right)\right)$$

• *θ* is the set of parameters of the generative model,
• *q*(*θ*) is an approximate posterior probability distribution,
• $D_{KL}\left(q\left(\theta \right)||p\left(\theta |y\right)\right)=\;\int q\left(\theta \right)\log\frac{q\left(\theta \right)}{p\left(\theta |y\right)}$ is a Kulback–Leibler divergence,
• *p*(*θ*|*y*) is the posterior distribution of the parameters.

We optimise *q*(*θ*) to approximate the posterior of the parameters of the model given the data, *p*(*θ*|*y*). As the LHS of Eq. (A.1) is constant and the last term *D*_*KL*_(*q*(*θ*)||*p*(*θ*|*y*)) is greater than 0 and equal to 0 when *q*(*θ*) = *p*(*θ*|*y*), maximising the integral will bring the approximate distribution as close as possible to the true posterior (as defined by the KL-divergence, *D*_*KL*_). The variational free energy, F, is defined as the integral on the right side. Note that it is equal to the difference between the expected energy of the approximate distribution and the entropy of the approximate distribution.

(A.2) $$F=\int d\theta \left[q\left(\theta \right)\ln p\left(y,\theta \right)-q\left(\theta \right)\ln q\left(\theta \right)\right]$$

We minimise Eq. (A.2) under the assumption of *n* independent Gaussian parameter distributions.

(A.3) $$q\left(\theta \right)=\prod_{i=1}^{n}q_{i}\left(\theta_{i}\right)$$

(A.4) $$q_{i}\left(\theta_{i}\right)=\;{\left(2\pi \right)}^{-\frac{p_{i}}{2}}{\left|\Sigma_{\theta ,i}\right|}^{-\frac{1}{2}}\exp(-\frac{1}{2}{\left(\theta_{i}-\mu_{i}\right)}^{T}\Sigma_{\theta ,i}^{-1}\left(\theta_{i}-\mu_{i}\right)$$

• *θ*_*i*_ is a subset of the set of parameters *θ* consisting of *p*_*i*_ parameters.

Define the following (energy) function,

(A.5) $$L\left(\theta \right)=\ln p\left(y,\theta \right)\;=\;\ln p\left(y|\theta \right)+\ln p\left(\theta \right)$$

The variational free energy can be written as follows,

(A.6) $$F=\int d\theta \left[q\left(\theta \right)\left(L\left(\theta \right)-\ln q\left(\theta \right)\right)\right]$$

Expand Eq. (A.5) in a Taylor series around the mean of *q*(*θ*) up to the second derivatives (Laplace approximation), and calculate its expectation over *q*(*θ*) (which are Gaussian distributions).

(A.7) $$L\left(\theta \right)=\;L\left(\mu \right)+\left(\partial_{\theta }L\right)\left(\theta -\mu \right)+\;\frac{1}{2}{\left(\theta -\mu \right)}^{T}\left(\partial_{\theta }^{2}L\right)\left(\theta -\mu \right)+O\left({\left(\theta -\mu \right)}^{3}\right)$$

(A.8) $$\int d\theta \left[q\left(\theta \right)L\left(\theta \right)\right]=\;L\left(\mu \right)+\;\frac{1}{2}\sum_{i}^{n}tr\left(\left(\partial_{\theta_{i}}^{2}L\right)\Sigma_{\theta ,i}\right)$$

The entropy of *q*(*θ*) is given by,

(A.9) $$\int d\theta \left[q\left(\theta \right)\ln q\left(\theta \right)\right]=\;-\frac{p}{2}\ln\left(2\pi \right)-\sum_{i}^{n}\frac{1}{2}\ln\left|\Sigma_{\theta ,i}\right|-\frac{p}{2}$$

• *p* is the dimension of the parameter space.

This gives a relatively simple expression for the free energy, parameterised by a set of Gaussian distributions.

(A.10) $$\begin{array}{l} F\left(\mu_{1},\ldots ,\mu_{n},\Sigma_{1,}\ldots ,\Sigma_{n}\right)\\ \;=\;L\left(\mu \right)+\;\frac{1}{2}\sum_{i}tr\left(\left(\partial_{\theta_{i}}^{2}L\right)\Sigma_{\theta ,i}\right)+\;\left[\frac{p}{2}\ln\left(2\pi \right)+\sum_{i}\frac{1}{2}\ln\left|\Sigma_{\theta ,i}\right|\right]+\frac{p}{2} \end{array}$$

At any local maxima of *F* the derivative of all variables will be zero. The 2nd and 4th term cancel when the above function is maximised over *μ*_*i*_ and Σ_*θ*,*i*_. Note that the same formula is usually derived by extremising *F* over the space of continuous differentiable functions using variational calculus and then reducing the space of approximating functions to Gaussian distributions. However, as the latter space is of finite dimension it is simpler to parameterise the variational free energy and maximise it over the parameterising scalar variables without needing to use variational calculus. At maxima:

(A.11) $$0=\frac{\partial F}{\partial \mu_{j}}=\;\frac{\partial L\left(\mu \right)}{\partial \mu_{j}}+\;\frac{1}{2}\sum_{i}tr\left(\left[\frac{\partial }{\partial \mu_{j}}\left(\partial_{\theta_{i}}^{2}L\right)\right]\Sigma_{\theta ,i}\right)$$

*L*(*θ*) is given by the following, under the assumption of Gaussian parameter priors and likelihood distribution:

(A.12) $$L\left(\theta \right)=-\frac{1}{2}\varepsilon^{T}{\Sigma_{m}}^{-1}\varepsilon -\frac{d}{2}\ln2\pi -\;\frac{1}{2}\ln\left|\Sigma_{m}\right|\;-\;\frac{1}{2}\;{\varepsilon^{\theta }}^{T}{P_{\theta }}^{-1}\varepsilon^{\theta }-\;\frac{p}{2}\ln2\pi -\frac{1}{2}\ln\left|P_{\theta }\right|$$

• *ε* = *y* − *G*(*θ*) is the prediction error.
• *G*(*θ*) is the function that maps parameters onto measurements (a linear approximation of which is derived from the Jacobian of the neural mass model).
• Σ_*m*_ is the covariance of the sampling errors.
• *d* is the dimension of the measurement space.
• *ε*^*θ*^ = *μ* − *ϑ*^*θ*^ is the difference between the posterior and prior mean.
• P_*θ*_ is the covariance of the prior distribution.

The first three terms on the RHS denote the log likelihood of the data given the parameters and the last three terms denote the log prior parameter distribution. The derivatives of *L*(*θ*) are given by (no summation over *i* in Eq. (A.14)):

(A.13) $$\begin{array}{l} \partial_{\mu_{i}}L\left(\mu \right)=-\frac{1}{2}{\left[\frac{\partial }{\partial \mu_{i}}\;G\left(\mu \right)\right]}^{T}{\Sigma_{m}}^{-1}\varepsilon -\;\frac{1}{2}\;\varepsilon^{T}{\Sigma_{m}}^{-1}\left[\frac{\partial }{\partial \mu_{i}}\;G\left(\vec{\mu }\right)\right]\;-\;\frac{1}{2}{\varepsilon^{\theta }}_{j}{\left\{{P_{\theta }}^{-1}\right\}}_{ji}\\ \;-\frac{1}{2}{\left\{P_{\theta }\right\}}_{ij}{\varepsilon^{\theta }}_{j} \end{array}$$

(A.14) $$\partial_{\mu_{i}}^{2}L\left(\mu \right)={\left[\frac{\partial }{\partial \mu_{i}}\;G\left(\mu \right)\right]}^{T}{\Sigma_{m}}^{-1}\left[\frac{\partial }{\partial \mu_{i}}\;G\left(\mu \right)\right]-\;{\left\{{P_{\theta }}^{-1}\right\}}_{ii}$$

Further, assuming that *G* is a linear mapping simplifies the estimate of the maxima of the free energy. The derivatives of *L*(*θ*) then take the form (no summation over *i* in Eq. (A.16)):

(A.15) $$\partial_{\mu_{i}}L\left(\mu \right)=-{\left\{G\right\}}_{ji}{\left\{{\Sigma_{m}}^{-1}\right\}}_{jk}{\left\{G\mu -y\right\}}_{k}\;-\;{\left\{{P_{\theta }}^{-1}\right\}}_{ij}{\left\{\mu -\vartheta^{\theta }\right\}}_{j}$$

(A.16) $$\partial_{\mu_{i}}^{2}L\left(\mu \right)=-{\left\{G\right\}}_{ji}{\left\{{\Sigma_{m}}^{-1}\right\}}_{jk}{\left\{G\right\}}_{ki}-\;{\left\{{P_{\theta }}^{-1}\right\}}_{ii}$$

Substituting these equations into Eq. (A.11) gives the following equation for the value of *μ*_*i*_ at the maximum of the free energy.

(A.17) $$0=-{\left\{G\right\}}_{ji}{\left\{{\Sigma_{m}}^{-1}\right\}}_{jk}{\left\{G\mu -y\right\}}_{k}\;-\;{\left\{{P_{\theta }}^{-1}\right\}}_{ij}{\left\{\mu -\vartheta^{\theta }\right\}}_{j}$$

This will consist of a set of *p* linear equations determining the position of the maxima of *F* and thus finding the means of the Gaussian distributions which optimally approximate the true posterior. The covariance can be estimated in exactly the same way.

(A.18) $$0=\frac{\partial F}{\partial \Sigma_{\theta ,i}}=\frac{\partial }{\partial \Sigma_{\theta ,i}}L\left(\mu \right)+\frac{1}{2}\left(\partial_{\mu_{i}}^{2}L\right)+\;\frac{1}{2}{\Sigma_{\theta ,i}}^{-1}$$

(A.19) $$\Sigma_{\theta ,i}=\;-{\left(\partial_{\mu_{i}}^{2}L\right)}^{-1}$$

Eq. (A.17) can then be rearranged as follows:

(A.20) $$-\left(G^{T}{\Sigma_{m}}^{-1}G+\;{P_{\theta }}^{-1}\right)\mu +G^{T}{\Sigma_{m}}^{-1}y+{P_{\theta }}^{-1}\vartheta^{\theta }\;=0$$

(A.21) $$\mu =\;{\left(G^{T}{\Sigma_{m}}^{-1}G+\;{P_{\theta }}^{-1}\right)}^{-1}G^{T}{\Sigma_{m}}^{-1}y+{\left(G^{T}{\Sigma_{m}}^{-1}G+\;{P_{\theta }}^{-1}\right)}^{-1}{P_{\theta }}^{-1}\vartheta^{\theta }$$

This equation gives the posterior means as a linear function of the data and the prior means. It is interesting to note that this equation has the same form as the equation for estimating hidden states in Kalman filtering:

(A.22) $$\mu =\;Ky+\left(I-KG\right)\vartheta^{\theta }$$

• *K* = P_*θ*_*G*^*T*^(*G*P_*θ*_*G*^*T*^ + Σ_*m*_) is the Kalman gain.

The covariance of the posteriors is updated using Eq. (A.19) which can be shown to be identical to the update of the covariance for Kalman filtering:

$$\Sigma =\;\left(I-KG\right){P_{\theta }}^{-1}$$

The same relations hold for non-linear *G* (if the 2nd and higher order terms are small compared to the first order term of the Taylor expansion of *G*) with the posterior mean and covariance given by

(A.23) $$\mu =\;\vartheta^{\theta }+\;{\left(\partial G^{T}\Sigma^{-1}\partial G+\;{P_{\theta }}^{-1}\right)}^{-1}\partial G^{T}\Sigma^{-1}\left[y-G\left(\mu^{\theta }\right)\right]$$

(A.24) $$\Sigma =\;{\left(\partial G^{T}{\Sigma_{m}}^{-1}\partial G+\;{P_{\theta }}^{-1}\right)}^{-1}$$

### A.2 Bayesian belief updating

#### A.2.1 Summary

Parameters vary slowly for seizures, allowing us to approximate them with stepwise constant functions. The algorithm described estimates the parameters in each window and then uses a model of how the parameters change from window to window. Here, this model was based on a random walk. This random walk might be itself governed by parameters; e.g., the amplitude of random fluctuations. These parameters can be estimated by maximising the free energy over individual windows and their trajectory (by supplementing the free energy to include the probability of any trajectory under a random walk model). See details below.

#### A.2.2 Bayesian belief updating

It is possible to infer the parameters of a system governed by slowly drifting parameters by repeated measurements if it can be assumed that the parameters are constant within the period of each measurement – known as an adiabatic approximation. The generative model will have a forward model describing how the parameters/states change over time and a measurement model describing how the parameters generate the data. The update equations described above can – with the prediction equations described in the main text – be used for updating slowly drifting parameters.

It is also possible to estimate the volatility R_*k*_ (see main text) from the data: new parameters, η_*k*_, can be introduced which parameterise R_*k*_ as follows.

$$R_{i}=\left(1+\eta_{i}\right)\Sigma_{\theta ,0}$$

where Σ_*θ*,0_ is the prior covariance of the parameters before observing the first epoch. The predicted prior covariance of the parameters in epoch *i* is then given by,

(A.26) $$P_{\theta ,i}=\;g\left(\eta_{i},\Sigma_{\theta ,i-1}\right)=\;\Sigma_{\theta ,i-1}+\left(1+\eta_{i}\right)\Sigma_{\theta ,0}$$

The energy, *L*(*θ*, η), is given by the following augmentation of Eq. (A.12):

(A.27) $$L\left(\theta ,\eta \right)=L_{\mathrm{old},i}\left(\theta \right)\;-\;\frac{1}{2}\;{\varepsilon^{\eta }}^{T}{P_{\eta ,i}}^{-1}\varepsilon^{\eta }-\;\frac{1}{2}\ln2\pi -\;\frac{1}{2}\ln\left|P_{\eta ,i}\right|$$

• *ε*^η^ = *ε*^η^ − *ε*_0_^η^ is the difference between the posterior and prior mean of the volatility parameters.
• P_*η*,*i*_ is the covariance of the prior distribution of the volatility parameters.

The corresponding free energy at time *i* is then given by:

(A.28) $$\begin{array}{l} F=F_{\mathrm{old}}\;-\;\frac{1}{2}\;{\varepsilon^{\eta }}^{T}{P_{\eta ,i}}^{-1}\varepsilon^{\eta }-\;\frac{1}{2}\ln2\pi -\;\frac{1}{2}\ln\left|P_{\eta ,i}\right|\;\\ +\;\frac{1}{2}tr\left(\left[\partial_{\eta_{i}}^{2}L\right]\Sigma_{\eta ,i}\right)\;+\frac{1}{2}\ln2\pi e+\frac{1}{2}\ln\left|\Sigma_{\eta ,i}\right| \end{array}$$

with derivatives:

(A.29) $$\begin{array}{l} \frac{\partial F}{\partial \eta_{i}}=-\left(\eta_{i}-\eta_{i}^{0}\right){P_{\eta ,i}}^{-1}\\ \;+\frac{1}{2}{\left(\theta_{i}-\mu_{i-1}^{1}\right)}^{T}g^{-1}\Sigma_{\theta ,0}g^{-1}\left(\theta_{i}-\mu_{i-1}^{1}\right)-\frac{1}{2}tr\left(g^{-1}\Sigma_{\theta ,0}\right)\\ \;+\frac{1}{2}tr\left(g^{-1}\Sigma_{\theta ,0}g^{-1}\Sigma_{\theta ,i}\right)\\ \;+\frac{3}{2}\Sigma_{\eta ,i}\left[{\left(\theta_{i}-\mu_{i-1}^{1}\right)}^{T}g^{-1}\Sigma_{\theta ,0}g^{-1}\Sigma_{\theta ,0}g^{-1}\Sigma_{\theta ,0}g^{-1}\left(\theta_{i}-\mu_{i-1}^{1}\right)\right)\\ \;-\left(\frac{1}{2}tr\left(g^{-1}\Sigma_{\theta ,0}g^{-1}\Sigma_{\theta ,0}g^{-1}\Sigma_{\theta ,0}\right)\right] \end{array}$$

This last equation is non-linear in η_*i*_ and can be difficult to solve. One way of estimating η_*i*_ is to keep η_*i*_ and Σ_*η*,*i*_ constant, perform the inversion of the non-volatility parameters and estimate the first and second derivative of the free energy over *η*_*i*_. One can then use the Newton–Raphson method to estimate the solution (roots) to the above equation and finally estimate the covariance. Formerly, define *f*(*η*_*i*_) as

$$f\left(\eta_{i}\right)=\;\frac{\partial F}{\partial \eta_{i}}$$

One can then find the root of the above equation using the Newton–Raphson method, giving an iterative sequence of updates for *η*_*i*_.

ηi'=ηi−fηif'ηi

where:

(A.30) $$\begin{array}{l} f'\left(\eta_{i}\right)=\;-{P_{\eta ,i}}^{-1}\\ -\;{\left(\theta_{i}-\mu_{i-1}^{1}\right)}^{T}g^{-1}\Sigma_{\theta ,0}g^{-1}\Sigma_{\theta ,0}g^{-1}\left(\theta_{i}-\mu_{i-1}^{1}\right)+\frac{1}{2}tr\left(g^{-1}\Sigma_{\theta ,0}g^{-1}\Sigma_{\theta ,0}\right)\\ -\;tr\left(g^{-1}\Sigma_{\theta ,0}g^{-1}\Sigma_{\theta ,0}g^{-1}\Sigma_{\theta ,i}\right)\;-\;6\Sigma_{\eta ,i}\;{\left(\theta_{i}-\mu_{i-1}^{1}\right)}^{T}g^{-1}\Sigma_{\theta ,0}g^{-1}\Sigma_{\theta ,0}g^{-1}\Sigma_{\theta ,0}g^{-1}\Sigma_{\theta ,0}g^{-1}\left(\theta_{i}\;-\mu_{i-1}^{1}\right)\\ +\;\frac{9}{4}\Sigma_{\eta ,i}tr\left(g^{-1}\Sigma_{\theta ,0}g^{-1}\Sigma_{\theta ,0}g^{-1}\Sigma_{\theta ,0}g^{-1}\Sigma_{\theta ,0}\right)\; \end{array}$$

at maxima

$$\Sigma_{\eta ,i}=\;-{\left[\frac{\partial^{2}}{\partial \eta_{i}^{2}}L\right]}^{-1}$$

This can be inserted into Eq. (A.28) to estimate the free energy for the posterior probability distributions with the estimated means, (*μ*, *η*) and covariances (Σ_*θ*,*i*_, Σ_*η*,*i*_).

Note that we have only maximised the free energy of a reduced model comprising the transition between epoch *i* − 1 and *i*. The approximate log evidence for any model can now be obtained by summing the free energy over all epochs.

### A.3 Practical aspects of Bayesian belief updating

#### A.3.1 Initialisation of the parameters of the model

A total of 30 parameters were included in the model and require initialisation prior to sequential inversion. This initialization was based on the first window of baseline activity. The spectral distribution of this window was used to estimate the values of the parameters using variational Laplace (Moran et al., 2008, Moran et al., 2011c). For the sequential inversion of seizure activity all but two parameters governing excitatory and inhibitory activity and 10 parameters governing the spectral input to the cortical region were kept constant. Moreover, the noise process (the volatility) of the inference scheme was optimised to maximise model evidence (i.e., variational free energy).
